# Functional Importance of Transient Receptor Potential (TRP) Channels in Neurological Disorders

**DOI:** 10.3389/fcell.2021.611773

**Published:** 2021-03-04

**Authors:** Kihwan Lee, Youn Yi Jo, Gehoon Chung, Jung Hoon Jung, Yong Ho Kim, Chul-Kyu Park

**Affiliations:** ^1^Gachon Pain Center and Department of Physiology, Gachon University College of Medicine, Incheon, South Korea; ^2^Department of Anesthesiology and Pain Medicine, Gil Medical Center, Gachon University, Incheon, South Korea; ^3^Department of Oral Physiology and Program in Neurobiology, School of Dentistry, Seoul National University, Seoul, South Korea; ^4^Program in Neurosciences and Mental Health, The Hospital for Sick Children, Toronto, ON, Canada

**Keywords:** TRP channels, neurological disorders, calcium homeostasis, Alzheimer’s disease, Parkinson’s disease, Huntington’s disease, amyotrophic lateral sclerosis, epilepsy

## Abstract

Transient receptor potential (TRP) channels are transmembrane protein complexes that play important roles in the physiology and pathophysiology of both the central nervous system (CNS) and the peripheral nerve system (PNS). TRP channels function as non-selective cation channels that are activated by several chemical, mechanical, and thermal stimuli as well as by pH, osmolarity, and several endogenous or exogenous ligands, second messengers, and signaling molecules. On the pathophysiological side, these channels have been shown to play essential roles in the reproductive system, kidney, pancreas, lung, bone, intestine, as well as in neuropathic pain in both the CNS and PNS. In this context, TRP channels have been implicated in several neurological disorders, including Alzheimer’s disease, Parkinson’s disease, Huntington’s disease, amyotrophic lateral sclerosis, and epilepsy. Herein, we focus on the latest involvement of TRP channels, with a special emphasis on the recently identified functional roles of TRP channels in neurological disorders related to the disruption in calcium ion homeostasis.

## Introduction

### TRP Channel Subfamily

Transient receptor potential (TRP) channels are classified into 28 members that function as a group of unique non-selective cation channels in mammals. TRP channels are conserved in yeast, invertebrates, and vertebrates and share a common three-dimensional structure with six transmembrane helical segments (S1–S6), two variable and intracellular amino (-NH_2_) and a carboxy (-COOH) terminal cytosolic domain, and the channel pore formed by S5 and S6, which allow transport of various ions including sodium (Na^+^), potassium (K^+^), calcium (Ca^2+^), and magnesium ions (Mg^2+^). Based on significant sequence homology and a common structure, TRP channels are divided into six subfamilies: TRPC (canonical), TRPM (melastatin), TRPV (vanilloid), TRPA (ankyrin), TRPP (polycystin), and TRPML (mucolipin). TRP subfamilies are divided into Group 1 (TRPC, TRPM, TRPV, and TRPA) and Group 2 (TRPP and TRPML) according to differences in their sequence and topology. Subfamilies of TRP channels are divided into groups and subtypes as represented in the phylogenetic tree in Supplementary Figure 1.

Transient receptor potential channels are ubiquitously expressed in many cell types (especially neurons and non-neuronal cells in the central nervous system, CNS) and tissues, including brain, kidney, pancreas, lung, bone, intestine, reproductive system as well as dorsal root ganglia (DRG) neurons in the peripheral nervous system (PNS). In addition, TRP channels are primarily expressed in plasma membranes that play critical roles in stimulus perception (i.e., thermosensation, mechanosensation, and chemosensation) and ion homeostasis (Nishida et al., 2006; Nilius and Owsianik, 2011).

Initially, TRP channels were shown to regulate cellular Ca^2+^ influx through the so-called store-operated channels (Nilius, 2004; Ramsey et al., 2006; Yazgan and Naziroglu, 2017). Several studies have shown that TRP channels regulate neuronal excitability, intracellular Ca^2+^ and Mg^2+^ homeostasis, as well as cell proliferation and differentiation (Nilius, 2004).

In addition to their physiological functions, TRP channels are known to contribute to various pathophysiological roles in neurological disorders of the CNS (Nilius, 2007; Colsoul et al., 2013; Takada et al., 2013).

### Neurological Disorders

Neurodegenerative diseases, such as Alzheimer’s disease (AD), Parkinson’s disease (PD), Huntington’s disease (HD), and amyotrophic lateral sclerosis (ALS) and epilepsy, collectively known as “neurological disorders,” have distinct pathologies and represent a significant medical burden in the modern world.

Alzheimer’s disease is the most common neurodegenerative disease in the world and is characterized by the accumulation of beta-amyloid (Aβ) plaques from amyloid precursor protein (APP) and hyperphosphorylated tau protein (Iqbal et al., 2010; Murphy and Levine, 2010). PD is also a common brain disorder, primarily characterized by a resting tremor, postural instability, rigidity, and bradykinesia caused by dopaminergic (DA) neuronal loss in the substantia nigra (SN) pars compacta (SNpc) (Michel et al., 2013; Kalia and Lang, 2015). HD is an inherited neurodegenerative disorder that causes cognitive deficits, emotional imbalance, and uncontrolled excessive motor movements caused by a CAG trinucleotide repeat expansion within the Huntingtin gene that leads to the synthesis of polyglutamine tracts (Kremer et al., 1994). ALS, also known as Lou Gehrig’s disease, is another fatal type of neurodegenerative motor disease characterized by the deterioration of motor neurons in the motor cortex, brainstem, and spinal cord that leads to impairments in voluntary movement (Guatteo et al., 2007). Finally, epilepsy is a neurological disorder characterized by recurrent epileptic seizures, abnormal brain activity, and unusual behavior.

Over the past few decades, enormous efforts have been made to unveil the pathogenesis of neurological disorders. For example, endoplasmic reticulum (ER) stress, also known as oxidative stress, which is caused by misfolded proteins and abnormal Ca^2+^ homeostasis, neuroinflammation, and mitochondrial dysfunction have been shown to lead to neuronal cell death. Most notably, Ca^2+^ regulation, which is involved in normal physiological functions such as neuronal survival, proliferation, differentiation, gene transcription, and exocytosis at synapses, has been shown to be dysregulated in various neurological disorders (Bojarski et al., 2008; Bezprozvanny, 2009; Grosskreutz et al., 2010; Surmeier et al., 2010; Wu et al., 2011; Nikoletopoulou and Tavernarakis, 2012).

Interestingly, several studies have reported a correlation between intracellular Ca^2+^ concentrations ([Ca^2+^]_i_) and other pathogenic mechanisms, including the imbalance between antioxidant function and reactive oxygen species (ROS) production (Gorlach et al., 2015) as well as mitochondrial dysfunction (Contreras et al., 2010; Pivovarova and Andrews, 2010).

In fact, exposure of neuronal cells to Aβ peptides, induces an elevation of [Ca^2+^]_i_ that leads to cell death as observed in *in vitro* experiments (Adhya and Sharma, 2019). Aggregation of α-synuclein, which is associated with the pathology of PD, can also induce neuronal cell death via the disruption of cellular Ca^2+^ homeostasis (Fonfria et al., 2005; Danzer et al., 2007). Furthermore, the polyglutamine-expanded huntingtin protein and mutant superoxide dismutase-1 (SOD1), which are implicated in the pathogenesis of HD and ALS, respectively, also disrupt cellular Ca^2+^ homeostasis (Giacomello et al., 2013; Barrett et al., 2014). The disruption of intracellular Ca^2+^ concentration in epilepsy induces ROS production, apoptosis, and caspase activation through mitochondrial oxidative stress (Yilmaz et al., 2011; Naziroglu and Ovey, 2015). Therefore, alleviating disturbances in Ca^2+^ homeostasis may represent a potential therapeutic target for the treatment of neurological disorders (Nilius, 2007; Colsoul et al., 2013; Takada et al., 2013).

## TRP Channels in Neurological Disorders

### TRP Channels in AD

**FIGURE 1:**
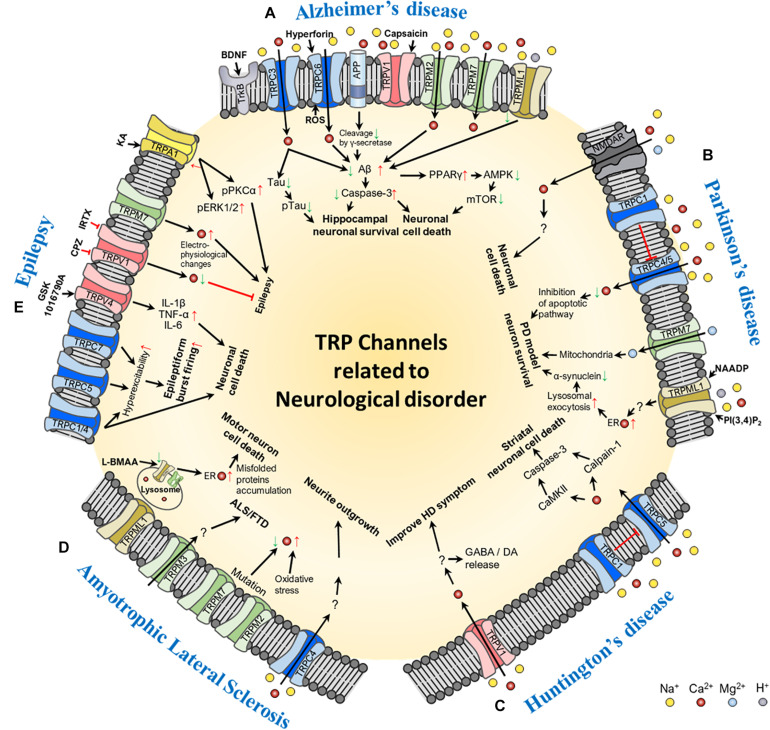
Schematic of the molecular mechanism of TRP channel-mediated pathogenesis of neurological disorders: **(A)** Alzheimer’s disease, **(B)** Parkinson’s disease, **(C)** Huntington’s disease, **(D)** Amyotrophic lateral sclerosis, and **(E)** epilepsy. In the figure, red arrows represent increase or up-regulation; green arrows, decrease or down-regulation; red thick bar, inhibition of the signaling pathway; and black arrows, activation of the signaling pathway. Ion channels involved in each of the neurological disorders and their transporting ions, channel agonists, and antagonists are also shown in the figure. *Abbreviations*: BDNF, brain-derived neurotrophic factor; ROS, reactive oxygen species; Aβ, beta-amyloid; PPAR, peroxisome proliferator-activated receptor gamma; AMPK, 5′ adenosine monophosphate-activated protein kinase; mTOR, mechanistic target of rapamycin; NMDAR, N-methyl-D-aspartate receptor; NAADP, Nicotinic acid adenine dinucleotide phosphate; PI(3,4)P_2_, phosphatidylinositol (3,4)-bisphosphate; PD, Parkinson’s disease; ER, endoplasmic reticulum; GABA, gamma-aminobutyric acid; DA, dopamine; ALS/FTD, amyotrophic lateral sclerosis patients with frontotemporal dementia; L-BMAA, L-beta-methylamino-L-alanine; IL, interleukin; TNF-α, tumor necrosis factor alpha; CPZ, capsazepine; IRTX, 5′-Iodoresiniferatoxin; KA, kainic acid; pPKCα, phospho-protein kinase C alpha; pERK1/2, phospho-extracellular signal-regulated kinase 1/2.

Importantly, a strong correlation between the pathological hallmarks of AD (Aβ accumulation and neurofibrillary tangles) and perturbed cellular Ca^2+^ homeostasis have been reported in AD patients as well as in animal and cell culture models of AD (Mattson and Chan, 2001). To date, TRPC1, TRPC3, TRPC6, TRPM2, TRPM7, TRPV1, TRPV4, TRPA1, and TRPML1 have been shown to be involved in AD (Figure 1A). TRPC1 is a member of the most prevalent TRPC channels in the brain and is linked to the store-operated Ca^2+^ channel-mediated Ca^2+^ entry (SOCE) channels. Interestingly, SOCE was reduced by downregulation of TRPC1 in astrocytes in APP knockout (KO) mice (Linde et al., 2011). Additionally, the alteration of the brain-derived neurotrophic factor-tropomyosin receptor kinase B-TRPC3 (BDNF-TrkB-TRPC3) signaling pathway led to hyperphosphorylation of tau protein caused by increased [Ca^2+^]_i_ levels in AD (Elliott and Ginzburg, 2006). Moreover, it has been reported that TRPC1 and TRPC3 are associated with caveolin-1, which is the main component of the plasma membrane caveolae that interacts with APP (Ikezu et al., 1998). Several studies have reported that TRPC6 in neurons promotes neuronal survival (Jia et al., 2007), synaptogenesis, and learning and memory (Zhou J. et al., 2008). Furthermore, in pharmacological studies using hyperforin, one of the main natural compounds of the medicinal plant Saint John’s wort that acts as an antidepressant drug (Zanoli, 2004) and TRPC6 activator (Tu et al., 2010), or tetrahydrohyperforin, a stable semisynthetic compound derived from hyperforin (Rozio et al., 2005), TRPC6 was shown to play a potential role in AD through the reduction of Aβ accumulation due to increased cerebrovascular P-glycoprotein (Brenn et al., 2014) and increased adult hippocampal neurogenesis and long-term spatial memory (Abbott et al., 2013), respectively. In contrast, AD-linked presenilin (PS)-2 mutants influenced TRPC6-mediated neurotoxic Ca^2+^ entry (Lessard et al., 2005), whereas TRPC6 was shown to be neuroprotective against AD through interaction with the cleavage of APP (Wang et al., 2015). In fact, a recent study observed that hyperforin induced activation of TRPC6, reduced Aβ levels, and improved mild cognitive impairment in AD models and that TRPC6 mRNA levels in the blood cells were reduced in AD patients (Lu et al., 2018).

While APP/PS1 transgenic (Tg) mice were observed to have age-dependent spatial memory deficits through TRPM2 channel activation by toxic oligomeric Aβ, genetic elimination of TRPM2 in APP/PS1 Tg mice ameliorated the synaptic loss and spatial memory deficits (Ostapchenko et al., 2015). It has been suggested that the TRPM2 channel may be a possible therapeutic agent of neuronal toxicity and memory impairment in AD. In contrast, TRPM7, which is another TRPM channel, plays an important role in not only inducing anoxic neuronal cell death by disrupting Ca^2+^ and Mg^2+^ homeostasis but also increasing susceptibility to degenerative processes (Aarts et al., 2003).

In recent studies of TRPV channels, the activity of TRPV1 was reported to reduce oxidative/nitrosative stress (Hofrichter et al., 2013; Jayant et al., 2016), rescue Aβ-induced degradation of hippocampal neurons (Balleza-Tapia et al., 2018), and increase levels of presynaptic synapsin I and postsynaptic density 93 (PSD93). In contrast, increased TRPV4 expression has been observed in the brain of aged rats (Lee and Choe, 2014), which is known to increase neuronal cell death via increased Ca^2+^ influx and ROS production (Hong et al., 2016). In APP/PS1 Tg/TRPA1 KO mice, the genetic loss of TRPA1 has been shown to exacerbate spatial learning and memory deficits, increase Aβ deposition, promote the release of pro-inflammatory cytokines such as interleukin (IL)-1β, IL-4, IL-6, and IL-10, and inhibit the activities of transcriptional factors NF-κB and nuclear factor of activated T cells (Lee et al., 2016).

Lastly, it has been shown that overexpression of TRPML1 is sufficient to rescue memory and cognitive deficits by diminishing neuronal apoptosis in APP/PS1 Tg mice (Zhang et al., 2017).

### TRP Channels in PD

TRPC1, TRPC3, TRPM2, TRPM7, and TRPV1 have been shown to be involved in PD (Figure 1B). The activation of TRPC1 channels has been reported to induce neuroprotection against apoptosis in SH-SY5Y neuroblastoma cells (Bollimuntha et al., 2006), as well as to regulate SOCE channels and reduce DA neuronal cell death in the SN of TRPC1 KO mice (Selvaraj et al., 2009, 2012). Consistent with these reports, decreased levels of TRPC1 have been detected in brain lysates from the SNpc of PD patients (Sun et al., 2017). In contrast to TRPC1 levels, TRPC3 levels are not altered in SNpc DA neurons in PD patients (Sun et al., 2017) although their levels are increased by the compensatory effect of decreased TRPC1 in 1-methyl-4-phenyl-1,2,3,6-tetrahyrdropyridine (MPTP)-induced PD-like conditions (Selvaraj et al., 2009). Constitutively active TRPC3 channels are known to express and regulate firing intensity and pattern in GABAergic neurons of the SN pars reticulata (SNpr) (Zhou F. W. et al., 2008). Therefore, this channel may also be involved in different brain subregions or pathophysiological mechanisms of PD.

Additionally, 1-methyl-4-phenylpyridinium ion (MPP^+^)-induced oxidative stress has been shown to increase intracellular Ca^2+^ influx via TRPM2 channel activity and promote DA neuronal cell death in the SNpc (Sun et al., 2018). Importantly, TRPM7 channels regulate Mg^2+^ homeostasis in cells, and increased concentrations of Mg^2+^ significantly inhibit MPP + -induced neurotoxicity by reducing the number of DA neurons and ameliorating the length of DA neurites (Hashimoto et al., 2008; Paravicini et al., 2012).

The activation of TRPV1 has also been shown to induce cell death in DA neurons, increase Ca^2+^ influx, and mediate mitochondrial disruption (Kim et al., 2005; Nam et al., 2015).

Furthermore, activation of TRPML1 evokes global ER Ca^2+^ release and Ca^2+^ influx (Kilpatrick et al., 2016) and results in upregulated lysosomal exocytosis, thus preventing α-synuclein accumulation in DA neurons (Tsunemi et al., 2019).

### TRP Channels in HD

TRPC1, TRPC5, and TRPV1 have been shown to be involved in HD (Figure 1C). Recently, it was shown that the expression level of endogenous TRPC1 was decreased in Q111 HD striatal cells compared to wild-type (Q7) cells (Hong et al., 2015). Furthermore, increased glutathionylation of TRPC5, activated by oxidants, leads to Ca^2+^-induced apoptosis of the striatal neurons in HD Tg mice (Hong et al., 2015). However, knockdown by siTRPC5 and inhibition of TRPC5 with ML204, a selective TRPC4 blocker produced by the Molecular Libraries Probe, produces a protective effect against oxidative stress in Q111 HD striatal cells and improves motor behavior in HD Tg mice (Miller et al., 2011; Hong et al., 2015, 2020a).

Moreover, it has been shown that administration of N-arachidonoylphenolamine (AM404), an inhibitor of endocannabinoid reuptake, has potential antihyperkinetic effects via the TRPV1 receptor using the 3-nitropropionic acid-induced HD model, suggesting that the activity of the TRPV1 channel may contribute to the motor dysfunction in HD patients (Lastres-Becker et al., 2003).

### TRP Channels in ALS

TRPC4, TRPM2, TRPM3, TRPM7, and TRPML1 have been shown to be involved in ALS (Figure 1D). The clinical symptoms of ALS overlap with those of Parkinsonism dementia complex (PDC), a neurodegenerative disorder characterized by symptoms of PD and dementia (Garruto, 2006; Hermosura and Garruto, 2007). While the pathogenesis of ALS/PDC is not fully understood, it is thought to be caused by two potential scenarios: (i) low levels of Ca^2+^ and Mg^2+^, which cause excess ROS production and cell death, and (ii) excess exposure to putative neurotoxin β-methylamino-L-alanine (L-BMAA), derived from the cycad plant, which causes an increase of [Ca^2+^]_i_ (Brownson et al., 2002).

A previous study demonstrated that the expression of TRPC4 is increased by nerve growth factor and dibutyryl-cAMP treatment in cultured DRG neurons. Conversely, inhibition of TRPC4 using a selective siRNA approach reduces neurite outgrowth in cultured DRG neurons (Wu et al., 2008). In ALS, reactive astrocytes accelerate nerve growth factor production (Pehar et al., 2004). In addition, a recent study demonstrated that ALS-resistant motor neurons from mutant SOD1 ALS models upregulate axonal outgrowth and dendritic branching (Osking et al., 2019). Taken together, these findings suggest that TRPC4 regulates DRG differentiation and plays a pivotal role in ALS.

In contrast, mutations in both TRPM2^P1018L^ and TRPM7^T1482I^ have been found in Guamanian ALS/PDC patients. Importantly, the TRPM2^P1018L^ variant was shown to attenuate oxidative stress-induced Ca^2+^ influx through inactivation of the channel (Hermosura et al., 2008). In contrast, the TRPM7^T1482I^ variant promotes an imbalance in Ca^2+^ and Mg^2+^ homeostasis (Hermosura and Garruto, 2007). TRPM3, which is in the same group as TRPM2, has also been considered as a possible candidate gene involved in the pathogenesis of ALS with frontotemporal dementia (Lee et al., 2003).

TRPML1 was expressed in cell lysosomes or in the endosomal membrane and in the main Ca^2+^-releasing channel used as a regulator of lysosomal storage via phosphatidylinositol 3,5-biphosphate (PI(3,5)P_2_) (Cheng et al., 2010; Li et al., 2013). In addition, TRPML1 also regulates the maintenance of lysosome homeostasis and accumulation of autophagy (Cheng et al., 2010; Curcio-Morelli et al., 2010; Morgan et al., 2011). Interestingly, PI(3,5)P_2_ levels are significantly impaired in some forms of ALS (Chow et al., 2009; Osmanovic et al., 2017). Moreover, in an experimental model of ALS/PDC, the expression of TRPML1 was shown to be reduced through autophagy leading to the loss of motor neurons. Additionally, with an L-BMAA-induced ALS mouse model, it was discovered that TRPML1 is downregulated, and autophagy is impaired in primary motor neurons, which leads to ER stress and neuronal cell death (Tedeschi et al., 2019).

### TRP Channels in Epilepsy

TRPC1, TRPC3-7, TRPM2, TRPM7, TRPV1, TRPV4, and TRPA1 have been shown to be involved in epilepsy (Figure 1E). As mentioned above, TRPC channels are not only known to play an important role in neuronal outgrowth and survival during brain development but are also believed to play a pivotal role in various epileptogenic processes. For example, the expression of TRPC1 is increased in cortical lesions of epilepsy patients and regulated by the mediation of astrocyte-induced epilepsy (Zang et al., 2015). Using a pilocarpine (muscarinic agonist)-induced status epilepticus (PISE) model, studies have shown that the genetic elimination of TRPC3 reduces the susceptibility of seizures to pilocarpine, while enhancing the expression of TRPC3 induces hyperexcitability and increases susceptibility to epileptiform activity in the cortex (Zhou and Roper, 2014; Phelan et al., 2017). In contrast, the expression of TRPC6 has been shown to be down-regulated in chronic epileptic rats, whereas the genetic ablation by siTRPC6 increases seizure susceptibility and seizure-induced neuronal damage in the dentate gyrus but not in CA1 and CA3 neurons of the hippocampus (Kim and Kang, 2015). In addition, in other members of the TRPC family, the genetic deletion of TRPC1/4 reduces seizure-induced neuronal cell death. Furthermore, TRPC5 KO mice exhibit significantly reduced seizures as well as minimal seizure-induced neuronal cell death in the CA1 and CA3 areas of the hippocampus (Phelan et al., 2013). Conversely, the TRPC7 channel plays an important role in spontaneous epileptiform bursting in the CA3, the reduction of which is correlated with a reduction in PISE in TRPC7 KO mice (Phelan et al., 2014).

The TRPM family is also involved in the pathogenesis of epilepsy. For example, TRPM2 channels are co-expressed with the EF-hand domain-containing protein 1 gene, which is related to an increased susceptibility to juvenile myoclonic epilepsy, and are regulated in the hippocampal neurons (Katano et al., 2012). Moreover, TRPM7 has been shown to be activated during epilepsy (Aarts and Tymianski, 2005). Importantly, genetic ablation of TRPM7 blocks the activation of a cation current, which is produced by oxygen-glucose deprivation (I_OGD_), and prevents ROS-mediated I_OGD_ activation (Aarts et al., 2003).

Although the TRPV1 channel is believed to play an essential role in the development of neurogenic pain and inflammation in the sensory neurons (Caterina and Julius, 2001; Julius and Basbaum, 2001), it is also expressed in other brain regions, including the cerebral cortex, hippocampus, cerebellum, thalamus, hypothalamus, striatum, midbrain, and amygdala (Cristino et al., 2006). Furthermore, increased expression of TRPV1 has been found in the hippocampus of rats and the dentate gyrus of mice with temporal lobe epilepsy as well as in the cortex of patients with temporal lobe epilepsy (Bhaskaran and Smith, 2010; Sun et al., 2013; Saffarzadeh et al., 2015). In fact, a recent study suggested that the activation of the TRPV1 channel may play a key role in the development of epilepsy (Naziroglu and Ovey, 2015). Using capsazepine, 5′-iodoresiniferatoxin, and resolvins, the authors showed that inhibition of the TRPV1 channel induced protective effects against epilepsy and epilepsy-induced Ca^2+^ entry in the hippocampal and DRG neurons (Naziroglu and Ovey, 2015).

Activation of TRPV3 by eugenol was shown to suppress epileptiform field potentials and decrease the amplitude of field postsynaptic potentials evoked in CA1 neurons of the hippocampus and the third layer of the neocortex (Muller et al., 2006). Another study using the PISE model of epilepsy found that activation of TRPV4 by the specific agonist GSK1016790A increased pro-inflammatory cytokines (TNF-α, IL-1β, and IL-6), while the inhibition of TRPV4 by HC-067047, a selective TRPV4 antagonist, significantly increased cell survival post status epilepticus (Wang et al., 2019).

Using a kainic acid-induced seizure model, TRPA1 was found to be upregulated while TRPV4 was not, which is contradictory to the earlier findings concerning TRPV4 (Hunt et al., 2012; Wang et al., 2019).

## Conclusion and Future Perspectives

In this review, we described the functional importance of TRP channels in the regulation of Ca^2+^ and oxidative stress responses as well as their contributions to neurological disorders, including AD, PD, HD, ALS, and epilepsy.

Overall, in addition to playing a broad range of physiological roles throughout the CNS and PNS, TRP channels also contribute to pathophysiology across a wide range of diseases and disorders through abnormalities in Ca^2+^ homeostasis. In the CNS, TRP channels are expressed in several brain regions (including the spinal cord) and have been shown to be key regulatory proteins involved in lipid metabolism, glucose homeostasis (Liu et al., 2009; Zhu et al., 2011), and the pathobiology of aforementioned neurological disorders.

Apart from their important role in neurological disorders of the CNS, TRP channels are also expressed in the neurons of the DRG, trigeminal ganglion, and sympathetic ganglion, and contribute to both normal and pathological sensory processing in the PNS (Lee et al., 2019). For example, TRP channels are known to be involved in diabetic peripheral neuropathy, chemotherapy-induced peripheral neuropathy, and autonomic neuropathy.

In light of the physiological and pathophysiological functions of TRP channels in both the CNS and PNS, we believe that they represent potential therapeutic targets for treating neurological disorders of the CNS as well as neuropathic pain in the PNS.

## Author Contributions

KL, YYJ, YHK, and C-KP contributed to the conception and design. KL and YYJ drafted the manuscript. KL, GC, JHJ, YHK, and C-KP revised the manuscript. All authors contributed to the article and approved the submitted version.

## Conflict of Interest

The authors declare that the research was conducted in the absence of any commercial or financial relationships that could be construed as a potential conflict of interest.

## Supplementary Material

Supplementary Figure 1 Summary of TRP subfamilies-mediated studies related to neurological disorders [modified from Nilius (2007), Hong et al. (2020b), Thapak et al. (2020)]. Different colors are used in the table for better classification and the gray boxes in the “Related Disease” column represent the roles of specific TRP subfamilies in each neurological disorders as reported in the published literature. *Abbreviations*: AD, Alzheimer’s disease; ALS, amyotrophic lateral sclerosis; AMG, amygdala; APP, amyloid precursor protein; BBB, blood-brain barrier; BG, basal ganglia; Bs, brainstem; CB, cerebellum; CC, corpus callosum; Cd, caudate; Cg, cingulate gyrus; CNS, central nervous system; CTX, cortex; DA, dopamine; DG, dentate gyrus; DRG, dorsal root ganglion; ER, endoplasmic reticulum; FB, forebrain; fCTX, frontal cortex; GABA, gamma-aminobutyric acid; GP, globus pallidus; HD, Huntington’s disease; HPC, hippocampus; HT, hypothalamus; IC, inferior colliculus; JME, juvenile myoclonic epilepsy; KA, kainic acid; LS, lateral septum; MB, midbrain; mFBb, medial forebrain bundle; mPFC, medial prefrontal cortex; mTOR, mechanistic target of rapamycin; n.d., not determined; OB, olfactory bulb; PD, Parkinson’s disease; PISE, pilocarpine-induced status epilepticus; PNS, peripheral nervous system; Pm, putamen; PS1, presenilin 1; RCh, retrochiasmatic area; SC, spinal cord; Sep, septum; SN, substantia nigra; SNpc, substantia nigra pars compacta; SNpr, substantia nigra pars reticulata; SOCE, store-operated calcium entry; Str, striatum; TH, thalamus; vS, ventral subiculum.
