# Supplementary figures and images for: Functional Importance of Transient Receptor Potential (TRP) Channels in Neurological Disorders
Source: Front Cell Dev Biol. 2021 Mar 4;9:611773. doi: 10.3389/fcell.2021.611773 (PMC7969799; doi:10.3389/fcell.2021.611773)

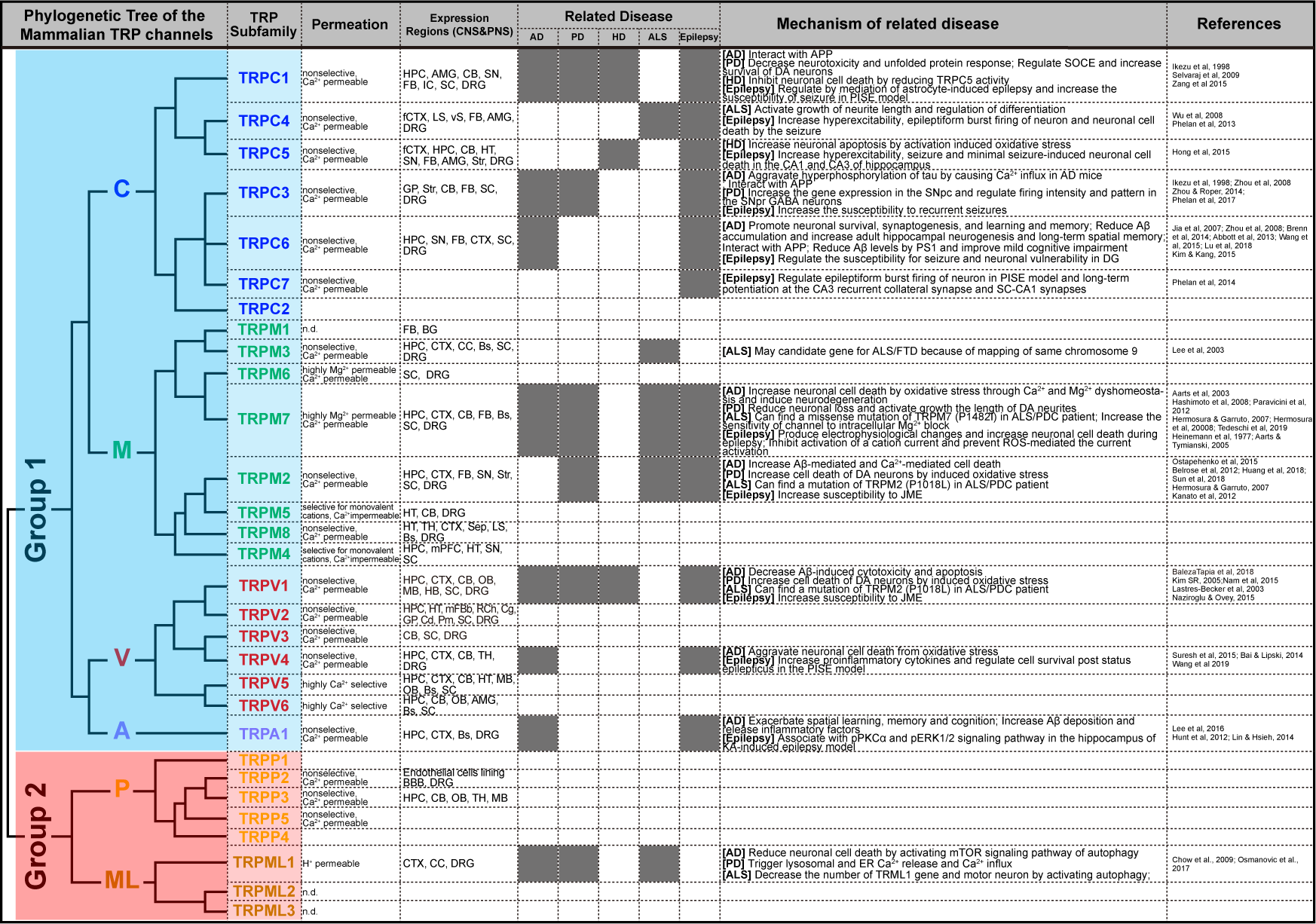

Supplement: Supplementary Figure 1 — Summary of TRP subfamilies-mediated studies related to neurological disorders [modified from Nilius (2007), Hong et al. (2020b), Thapak et al. (2020)]. Different colors are used in the table for better classification and the gray boxes in the “Related Disease” column represent the roles of specific TRP subfamilies in each neurological disorders as reported in the published literature. Abbreviations: AD, Alzheimer’s disease; ALS, amyotrophic lateral sclerosis; AMG, amygdala; APP, amyloid precursor protein; BBB, blood-brain barrier; BG, basal ganglia; Bs, brainstem; CB, cerebellum; CC, corpus callosum; Cd, caudate; Cg, cingulate gyrus; CNS, central nervous system; CTX, cortex; DA, dopamine; DG, dentate gyrus; DRG, dorsal root ganglion; ER, endoplasmic reticulum; FB, forebrain; fCTX, frontal cortex; GABA, gamma-aminobutyric acid; GP, globus pallidus; HD, Huntington’s disease; HPC, hippocampus; HT, hypothalamus; IC, inferior colliculus; JME, juvenile myoclonic epilepsy; KA, kainic acid; LS, lateral septum; MB, midbrain; mFBb, medial forebrain bundle; mPFC, medial prefrontal cortex; mTOR, mechanistic target of rapamycin; n.d., not determined; OB, olfactory bulb; PD, Parkinson’s disease; PISE, pilocarpine-induced status epilepticus; PNS, peripheral nervous system; Pm, putamen; PS1, presenilin 1; RCh, retrochiasmatic area; SC, spinal cord; Sep, septum; SN, substantia nigra; SNpc, substantia nigra pars compacta; SNpr, substantia nigra pars reticulata; SOCE, store-operated calcium entry; Str, striatum; TH, thalamus; vS, ventral subiculum. [file Image_1.tif]
